# Acknowledgment to the Reviewers of *BioTech* in 2022

**DOI:** 10.3390/biotech12010011

**Published:** 2023-01-17

**Authors:** BioTech Editorial Office

**Affiliations:** MDPI AG, St. Alban-Anlage 66, 4052 Basel, Switzerland

High-quality academic publishing is built on rigorous peer review. *BioTech* was able to uphold its high standards for published papers due to the outstanding efforts of our reviewers. Thanks to the efforts of our reviewers in 2022, the median time to first decision was 18 days and the median time to publication was 43 days. Regardless of whether the articles they examined were ultimately published, the editors would like to express their appreciation and thank the following reviewers for the time and dedication that they have shown *BioTech*:
Abhinav JainLarry J. GrabauAbhiram ArunkumarLászló Csaba MangelAchchuthan ShanmugasundramLaura VitaleAgnieszka StecLeonidas AkritidisAlain Favre-RéguillonLi-Wei ChangAlbert BordonsLucjusz ZaprutkoAlessio TallaritaLucy CarterAlex Omo IbhadonMagdalena GawłowskaAna Rita Xavier De Jesus GameiroMagdalena RyśAndrzej KasperskiMałgorzata OkręglickaAngela KranzMarina Svetec MiklenićAnna DrabczykMartina KroppAnna-Maria BarciszewskaMelinda SzilágyiAntonino VallesiMichael A. KouritzinAntonio CicchellaMiguel Toribio-MateasArtem RozhinMohamed Ewis AbdelazizAshraf VirmaniMohammad FaizanAvi MagidMustafa OzilgenAwdhesh MishraNadezhda SachivkinaAzza MohamedNataša KnapBarbara WlodarczykNguyen Quoc Khanh LeBernd SchöllhornNicco KrezdornCarsten NachtigallNick KouttabCaterina BuffoneNihad AdnanChengfei YanOgueri NwaiwuCherng-Yuan LinOsvin ArriagadaChung-Hsin WuPengwei LuChunxu TangPiotr RybarczykCoco C. H. De KoningPlacido BruzzanitiDamian Piotr MuniakPrakash BhuyarDaniel J. SmitPriyanka KumariDaniela PintoRahul GauttamDavid PadrosaRaimondo GaglioDavid Rodríguez-TemporalRodrigo ValenzuelaDavide PapurelloRoger M. LeBlancDhanesh Sivadasan BinduRuosen XieDilara R. MaslennikovaRuoxi ZhangDimitris A. GougoulisRuslan KalendarDisha MalaniSadaf JahanDorota Rutkowska-ZbikSaeid Najafi FardDulce Maria Palmerin-CarreñoSajeevan Radha SivarajanEleftherios TouloupakisSameera AbeyrathnaElżbieta Łodyga-ChruścińskaSamira TarashiElżbieta WołejkoSanbo QinEngy ElekhnawySandra HilárioFelice MastroleoSantosh A. MisalFrancisco Lázaro-DiéguezSashi DebnathGajanan ShelkarScott M. HoltGalina RalduginaSofia AgriopoulouGarry WongSonia MediouniGeorge P. LomonossoffSorin HostiucGiuseppe BadagliaccaSumbul SaeedHanseob JeongSusumu MitsuyamaHarifara RabemanolontsoaSyed Husain Mustafa RizviHimanshu BatraTadeusz KowalskiHuajiang HuangThanassis SpeliotisHumberto BizzoThomas D. HorvathIsabel Maria Gonzalez PadillaThomas R. JackIuliana PopescuTill BraunschweigIvan Ivanovich StoikovUmberto LuciaJ. Michael SorrellValeria BarresiJianli TaoValeria PistorioJianming LiValerio GiacconeJin-Ho YunVenkata MallampalliJinli WangVeronica SpinelliKai Cheng HsuVictor BanerjeeKai ZhaoVida JuozaitienėKaitlin A. PruskowskiWanda MączkaKapil D. PandeyWei ShaoKaruna DixitWei-Biao LiaoKasidid RuksakietXiaolong LiKhac-Minh ThaiXiaolu LiuKhalid A. KhalidXiaoying KongKi Hyun NamXie BingKoichi YoneyamaYan QinKrista MorleyYaodong GuKyung-Yong ChungYoshinori OkamotoLalit BatraYunzhou Li


